# Combined EEG and immersive virtual reality unveil dopaminergic modulation of error monitoring in Parkinson’s Disease

**DOI:** 10.1038/s41531-022-00441-5

**Published:** 2023-01-13

**Authors:** R. Pezzetta, D. G. Ozkan, V. Era, G. Tieri, S. Zabberoni, S. Taglieri, A. Costa, A. Peppe, C. Caltagirone, S. M. Aglioti

**Affiliations:** 1 Laboratory of Cognitive and Translational Neuroimaging, IRCCS San Camillo Hospital, 30126 Venice, Italy; 2 IRCCS Fondazione Santa Lucia, 00179 Rome, Italy; 3 Department of Psychology, University of Rome “La Sapienza”, 00185 Rome, Italy; 4 Virtual Reality Lab Unitelma Sapienza, 00161 Rome, Italy; 5 Niccolò Cusano University, 00166 Rome, Italy; 6 Center for Life Nano- & Neuro-Science, Fondazione Istituto Italiano di Tecnologia (IIT) and Sapienza University of Rome, 00161 Rome, Italy

**Keywords:** Parkinson's disease, Human behaviour

## Abstract

Detecting errors in your own and others’ actions is associated with discrepancies between intended and expected outcomes. The processing of salient events is associated with dopamine release, the balance of which is altered in Parkinson’s disease (PD). Errors in observed actions trigger various electrocortical indices (e.g. mid-frontal theta, error-related delta, and error positivity [oPe]). However, the impact of dopamine depletion to observed errors in the same individual remains unclear. Healthy controls (HCs) and PD patients observed ecological reach-to-grasp-a-glass actions performed by a virtual arm from a first-person perspective. PD patients were tested under their dopaminergic medication (on-condition) and after dopaminergic withdrawal (off-condition). Analyses of oPe, delta, and theta-power increases indicate that while the formers were elicited after incorrect vs. correct actions in all groups, the latter were observed in on-condition but altered in off-condition PD. Therefore, different EEG error signatures may index the activity of distinct mechanisms, and error-related theta power is selectively modulated by dopamine depletion. Our findings may facilitate discovering dopamine-related biomarkers for error-monitoring dysfunctions that may have crucial theoretical and clinical implications.

## Introduction

The progressive degeneration of dopaminergic neurons in the substantia nigra pars compacta that characterises Parkinson’s disease (PD) causes alterations in a complex circuit involving subcortical and cortical (mainly frontal and cingulate) regions^1–4^, leading to motor symptoms and higher-order cognitive function deficits^5,6^. Deficits have been described in performance and error monitoring^7–9^, planning and initiating voluntary movements^10^, conflict monitoring^11^, conflict suppression^12^, action inhibition^13,14^, motor interaction^15^, and reward-based learning^16^. Studies on dopamine’s influence on cognitive functions also indicate a role in regulating predictive processes^17–20^.

Making an error triggers specific electroencephalogram (EEG) signatures such as mid-frontal theta increases, delta power increases, and error-related negativity and positivity^21–24^. While smaller in amplitude and higher in latency^25^, similar signatures are also triggered when observing action errors^26^. Specifically, observing errors by others causes increased mid-frontal theta and frontal error-related negativity (oERN) and positivity (oPe). These changes are seen when observing an error committed by another^27^, by a partner during motor interactions^28,29^, and by an embodied virtual arm observed from a first-person perspective^30,31^ (1PP). The observation-related error-monitoring components show a smaller amplitude and delayed latency than the execution-related components^25^. Throughout this article, we will use Pe and ERN to indicate error-execution-related positivity and error-negativity findings, respectively, and oPe and oERN to indicate error-observation-related findings.

Increasing neuroanatomical and functional evidence suggests that the prefrontal cortex, including the dorsal anterior cingulate cortex (ACC), generates error-related mid-frontal theta and ERN as a low-level mismatch between actual and expected responses^22,32–35^. Conversely, Pe’s origin is less clear. It has been associated with the engagement of different areas, such as the anterior parts of the ACC^36^, posterior parts of the cingulate^37^ and insular^38^ cortices, which have also been associated with the salience network^8,39,40^. Pe/oPe appear to signal the awareness of an error following motivational and affective events and may be associated with post-error adaptation^41,42^.

The distinct cortical involvement of these error-related responses is consistent with evidence suggesting the existence of independent mechanisms underlying the error-monitoring system^43,44^ that appear to rely on distinct neurotransmitters^36,45^. Overbeek, Nieuwenhuis & Ridderinkhof (2005) and other pharmacological studies^46^ have shown that the mid-frontal cortex is densely targeted by ascending dopaminergic projections from the ventral tegmental area^47^ (VTA). These might affect mid-frontal theta and ERN amplitude but not the Pe and error-related delta activity, which have been associated with locus coeruleus-norepinephrine system activity^48–50^.

The VTA’s level of dopaminergic neuron degeneration might result in different disease progression severity and non-motor function alterations^51^. Indeed, studies investigating the effects of neurochemical lesions in the VTA, a major source of dopaminergic projections to the forebrain, in rats found a 30–46% reduction in dopamine that did not alter P3-like potentials in intracranial recordings^52,53^. Despite a few studies with similar findings, the evidence remains unclear since no study has investigated the dopaminergic influence on different error-monitoring processes. Therefore, testing PD patients under dopaminergic medication (on-condition) and after dopaminergic withdrawal (off-condition) could be an ideal model for exploring dopamine’s selective influence on different EEG error-monitoring markers.

While a few EEG studies have reported reduced ERN and theta amplitude during action execution in PD patients, others reported mixed Pe and oPe results^8,9^. It should be noted that only two studies used the time-frequency (TF) approach^54,55^, and only one found no difference in conflict-related theta due to dopaminergic medication^55^. Therefore, it remains unclear whether dopamine balance is necessary for human mid-frontal theta activity during error monitoring.

In addition to theta, other frequencies are implicated in error-monitoring processes and PD. Delta (2–4 Hz) frequencies were associated with the Pe response, with erroneous actions characterised by a positive deflection in the time domain and an enhanced delta power^47,56^. However, several studies found beta alteration patterns in patients with dopaminergic deficiency^57^, including an exaggerated burst of beta oscillations, which has often been associated with motor impairments in this population.

Here, we investigated how dopamine balance influences error-monitoring mechanisms by recording EEGs in the same PD patients while on- and off-condition and in healthy controls. Participants were immersed in a virtual scenario and passively observed a virtual arm executing correct or incorrect actions from a 1PP. This approach proved adept at inducing the illusion of ownership over the virtual body, allowing us to investigate error processing in highly realistic circumstances^30,31,58^. Moreover, exploring action processing without overt movements allowed us to control any confounding due to interindividual differences in task difficulty or response speed that might occur between patients.

We hypothesised that distinct error processes co-exist^43^ and that off-condition patients would show specific alterations in the electrocortical error processing markers purportedly modulated by dopamine (i.e. mid-frontal theta) without affecting markers that are less related to it (i.e. oPe and delta^56,59^). Specifically, we expected off-condition PD patients to show a different theta-response (i.e. less theta activity in response to errors) than healthy controls (HCs), while the on-condition PD patients would have a similar activity to the HCs due to their dopaminergic treatment. We also explored whether the dopaminergic treatment produced a difference in theta activity between on- and off-conditions. We expected no difference in the oPe response among the three groups since this process appears not to be influenced by dopamine balance^59^. Furthermore, relationships between EEG motor error-observation markers and clinical scales assessing motor disability and disease progression were explored to highlight potential associations between clinical deficits and EEG states during different dopaminergic conditions.

Previous studies have shown the relationship between frontocentral theta, executive functions, and working memory abilities^60,61^. In this study, we explored the relationship between differential theta activity and tests assessing executive functions in PD patients. Specifically, we used two tests previously used in error-monitoring studies in the neurological and psychiatric populations^62,63^. We expected relationships between differential theta activity and executive function’ tests in PD since theta activity is a neural signature of cognitive control^4^. Furthermore, many studies have shown how PD patients without dopaminergic treatment tend to show exaggerated beta activity bursts during resting-state^64^ and cognitive control tasks^65^. To date, no study has investigated beta-band activity during error observation in the context of dopaminergic treatment. However, since previous studies have shown that dopamine improves neural communication associated with beta-band activity^66^, we expected to observe beta frequency modulation only in off-condition PD patients compared to HCs since beta activity will be higher in PD patients without dopaminergic treatment^57^.

## Results

### Clinical deficits relative to dopamine states inferred from UPDRS III and H&Y scales

Consistent with the expected beneficial dopamine effect for extrapyramidal symptoms, the UPDRS III scores decreased significantly between off-condition (M = 37.21, SD = 10.04) and on-condition (M = 17.67, SD = 6.80) PD patients (*t*_(13)_ = 5.39, *p* = 0.0001). H&Y scores ranged between 2 and 2.5 for on- and off-condition PD patients, except for one with a score of 3 in the off-condition. This finding suggests that all PD patients included in the study had bilateral disease since scores <1.5 indicate unilateral involvement only. Changes in H&Y scale values with different dopamine levels indicates a similar effect with significantly higher values in off-condition (M = 2.32, SD = 0.32) than on-condition (M = 2.08, SD = 0.18) PD patients (*t*_(13)_ = 2.78, *p* = 0.016; Table 1b). One patient was excluded from this analysis because their off-condition evaluation was missing. No significant correlation was found between UPDRS, H&Y, and EEG signals (theta, oPe).

**Table 1:** Demographic and clinical data.

(a)			
	PD (*N* = 15)	HCs (*N* = 14)	
	Mean ± SD	Mean ± SD	*p* (<0.05)
Sex	10 M, 5 F	9 M, 5 F	n.s. (0.89)
Age	69.93 ± 8.75	69.57 ± 6.06	n.s. (0.90)
Education	11.60 ± 4	13. 07 ± 2.58	n.s (0.25)
MMSE	29.13 ± 0.64	29.07 ± 1	n.s. (0.84)
MMPSE	29.79 ± 2.29	—	
LEDD	632.83 ± 186.55	—	

(b)			
	PD on (*N* = 15)	PD off (*N* = 15)	
	Mean ± SD	Mean ± SD	*p* (<0.05)
UPDRS-III	17.67 ± 6.80	37.21 ± 10.04	s. (0.0001)
H&Y	2.08 ± 0.18	2.32 ± 0.32	s. (0.02)

(a) Summary of demographics and clinical scores for the PD and HCs groups. age: age in years, education: education in years, *MMSE* mini-mental state examination, *MMPSE* mini-mental Parkinson state examination, *LEDD* levodopa equivalent daily dose.

(b) Summary of motor scale scores (unified PD rating scale, section III [UPDRS-III]) and disease progression (Hoehn and Yahr [H&Y] scale) of PD patients tested on- (with dopaminergic medication) and off-condition (without dopaminergic medication). *n.s*. non-significant, *s* significant.

### Correlations between theta activity and executive function abilities

No significant correlation survived multiple comparison correction.

## EEG

### Time-domain analysis

#### Cluster-based statistics

**Fig. 1:**
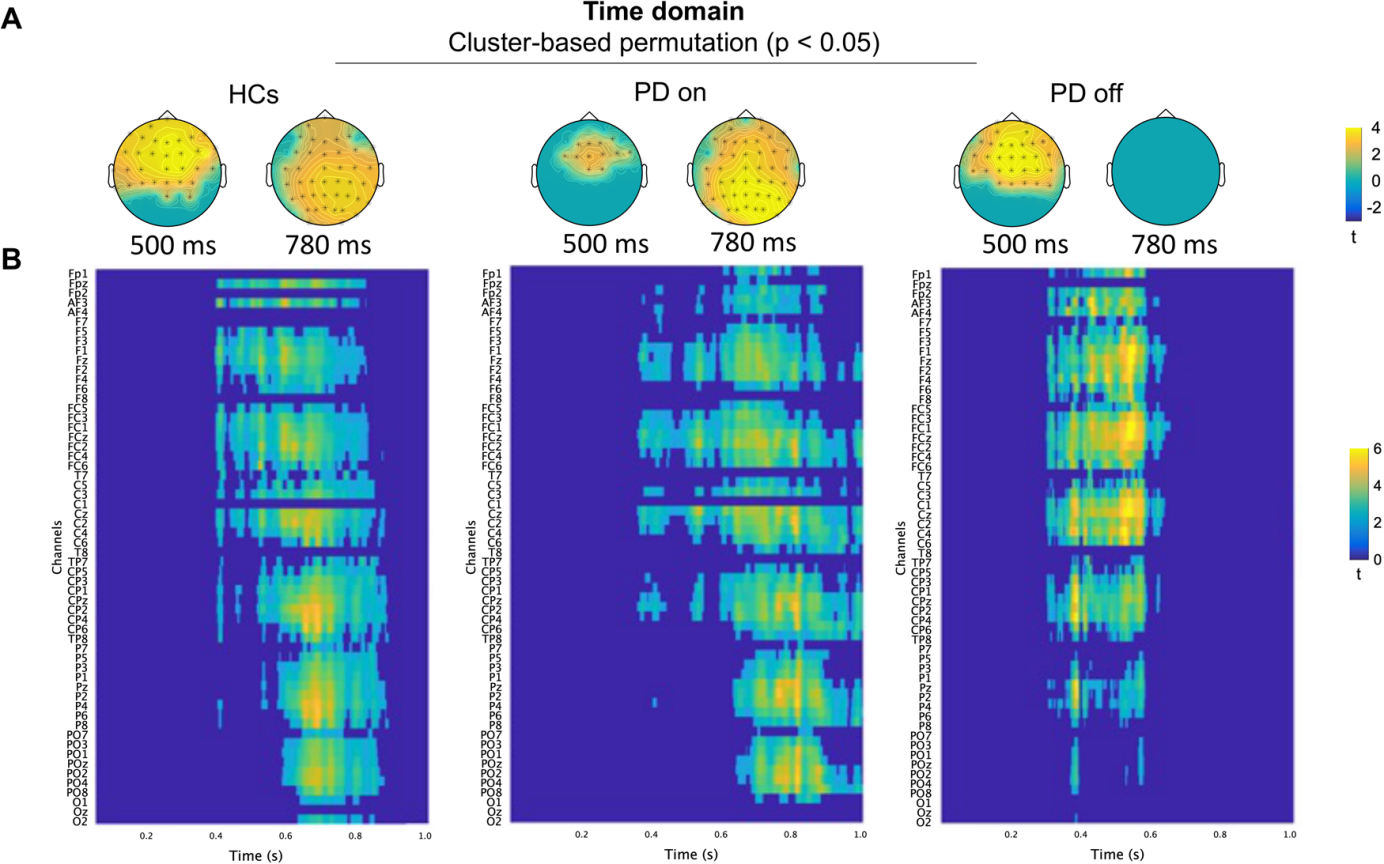
Cluster-based permutations in the time domain for each group. **A** Scalp representation of the cluster-based permutations (dependent sample *t*-tests with cluster-correction *p* < 0.05) of incorrect vs correct action extracted at two representative time points inside the window of interest. **B** Channel (*y*-axis) vs time (*x*-axis) representation of the cluster-based permutation for incorrect vs correct actions in the three groups; 0 ms corresponds to the avatar’s arm-path deviation, and the movements end at ~300 ms. For a larger visualisation of (**B**), please see https://osf.io/z9rbu/files/osfstorage (Figures section).

We found significant differences in the within-group analysis for all three groups but with different spatial distributions. A significant difference was found between correct and incorrect actions in the HCs (*p* = 0.0079, range 360–876 ms). Similarly, the cluster-based permutation revealed a difference between on-condition (*p* = 0.0020, range 380–1000 ms) and off-condition (*p* = 0.0079, range 300–634 ms) PD patients. Off-condition PD showed greater voltage in frontocentral rather than parietal electrodes after observing erroneous actions, with increased time-limited activity (Fig. 1). Cluster comparisons between groups did not show significant differences in the 0–1000 ms window.

#### Single-electrode statistics

**Fig. 2:**
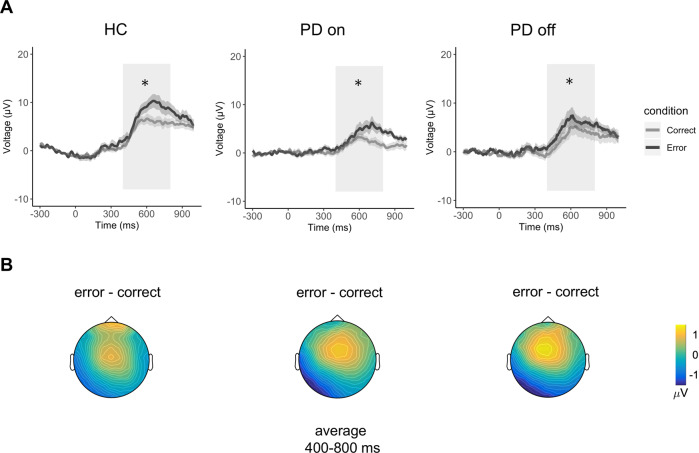
Electrophysiological results in the time domain for each group (ERPs). **A** Grand average oPe waveforms at electrode Pz. The end of the avatar’s movement is at 0 ms. Lighter colours denote the standard error of the mean. The light grey rectangle demarks the analysis interval window. **B** Graphical representation of the voltage distribution. Values represent incorrect minus correct actions.

An oERN analysis was not performed as a clear peak was not found on visual inspection. One possible interpretation of this negative finding is provided in the Discussion. Traditional oPe analyses (400–800 ms) showed that all groups differed significantly between correct and incorrect actions (Fig. 2). ANOVA comparing HCs and on-condition PD showed a significant condition effect (*F*_(27,1)_ = 8.77, *p* = 1.05 × 10^−6^; *η*^2^*p* = 0.52), with generally greater oPe values in HCs than in on-condition PD patients, and significantly greater oPe for incorrect than correct actions (*F*_(27,1)_ = 29.11, *p* = 0.0060; *η*^2^*p* = 0.25). There was also a significant condition vs electrode interaction (*F*_(27,1)_ = 11.96, *p* = 0.0010; *η*^2^*p* = 0.31). Post hoc analyses with multiple testing corrections showed that incorrect actions caused increased FCz and Pz electrode activity compared to correct actions.

The within-group ANOVA comparing on- and off-condition PD showed a significant condition effect (*F*_(14,1)_ = 17.73, *p* = 0.0008; *η*^2^*p* = 0.56) and condition vs electrode interaction (*F*_(14,1)_ = 14.15, *p* = 0.0020; *η*^2^*p* = 0.50). Similarly, post hoc analyses of the interaction showed that incorrect actions caused increased FCz and Pz electrode activity compared to correct actions. Finally, the ANOVA between HCs and off-condition PD again showed a significant condition effect (*F*_(27,1)_ = 23.68; *p* = 4.35 × 10^−5^; *η*^2^*p* = 0.47) and condition vs electrode interaction (*F*_(27,1)_ = 19.4, *p* = 0.0001; *η*^2^*p* = 0.42), with both FCz and Pz electrodes showing greater activity with incorrect than correct actions. However, no significant group vs condition interaction was found.

The ANOVAs on the differential (incorrect minus correct) waves showed only an electrode effect, with greater amplitude on FCz than Pz in all groups: HCs vs off-condition PD (*F*_(27,1)_ = 19.48, *p* = 0.0001; *η*^2^*p* = 0.42), on- vs off-condition PD (*F*_(14,1)_ = 14.15, *p* = 0.0020; *η*^2^*p* = 0.5000), and HCs vs on-condition PD (*F*_(27,1)_ = 11.96, *p* = 0.0018; *η*^2^*p* = 0.31).

No group effect was identified in any analysis. The absence of a group effect but the presence of a condition effect in the differential wave analyses is consistent with the absence of inter-group differences in the cluster-based permutation analyses, suggesting that all three groups had a consistently greater amplitude in their oPe responses to incorrect than correct actions. Also, Bayesian paired sample *t*-tests between on- and off-condition PD showed moderate evidence favouring the null hypothesis (BF_10_ = 0.26), while independent-sample *t*-tests between HCs and on- or off-condition PD showed anecdotal evidence supporting the null hypothesis (BF_10_ = 0.41 and BF_10_ = 0.39, respectively).

The effect sizes (*η*^2^*p*) for the main group effect did not show appreciable differences in magnitude when comparing oPe between HCs and off-condition PD (*p* = 0.7831; *η*^2^*p* < 0.01), HCs and on-condition PD (*p* = 0.6319; *η*^2^*p* = 0.01), or on- and off-condition PD (*p* = 0.8261; *η*^2^*p* < 0.01).

### TF domain analysis

#### Theta (4–8.1 Hz)

##### Cluster-based statistics

We found significant differences between incorrect and correct actions in the HCs (*p* = 0.0019, range 208–888 ms) and on-condition PD (*p* = 0.011, range 0–648 ms), with greater theta activity for incorrect actions and a most pronounced difference over the central areas. However, there was no significant difference between incorrect and correct actions in off-condition PD. HCs and off-condition PD patients differed significantly (*p* = 0.0319, range 392–792 ms), with the most pronounced difference in the frontal and posterior areas. No other significant differences between groups were found.

##### Single-electrode statistics

**Fig. 3:**
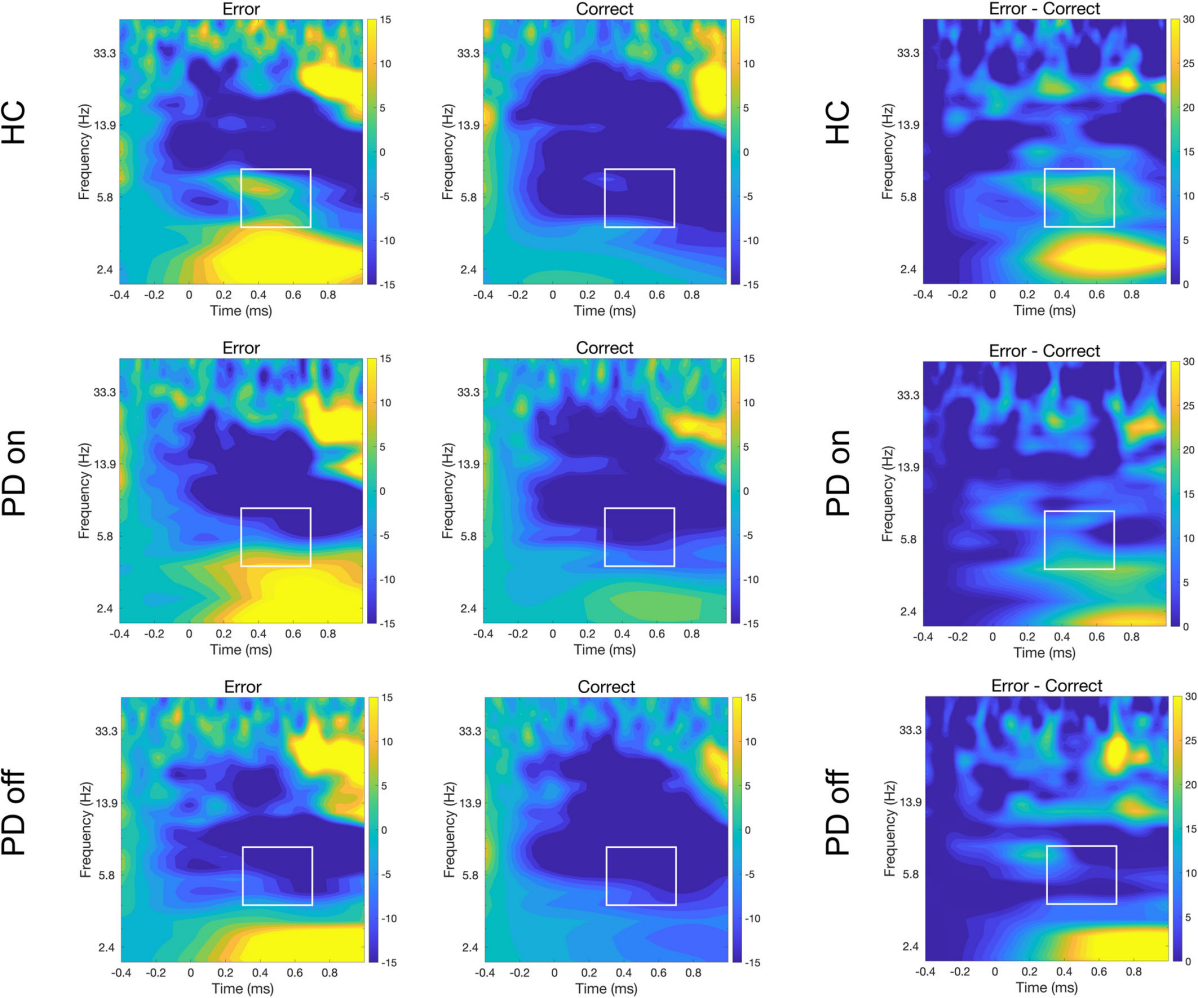
TF representation of relative power change (in %) with respect to baseline for incorrect and correct actions. The end of the avatar’s arm-path deviation is at 0 ms. Incorrect and correct plots for electrode FCz between 1 and 50 Hz are shown for each group. Differential plots (erroneous − correct actions) are provided in the third column. The white rectangles demark the a priori chosen window of interest between 300–700 ms and 4–8.1 Hz, which were the values used for statistical analyses.

**Fig. 4:**
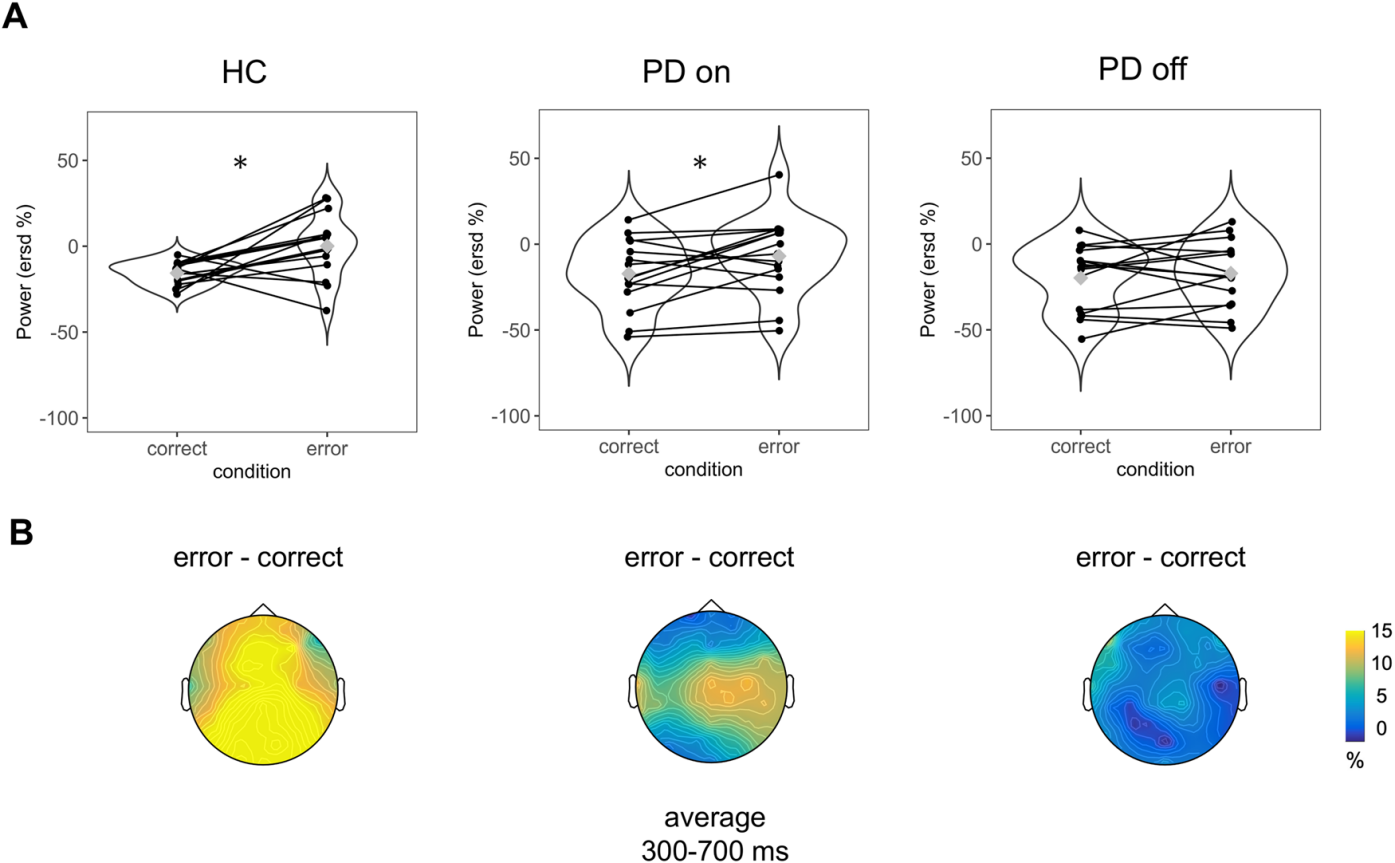
Graphical representation of theta power (4–8.1 Hz) in the three groups on electrode FCz. **A** Violin plots represent theta activity with correct and incorrect actions. *Y*-axes represent theta power expressed in relative power change (in %). Grey diamonds in the violin plots denote the mean value; black lines connect individual subject observations (i.e. black points) in the two conditions. **B** Graphical representation of voltage distribution. The values indicate the difference between incorrect and correct actions.

The ANOVAs comparing HCs and on-condition PD showed a main condition effect (*F*_(27,1)_ = 16.54, *p* = 0.0003; *η*^2^*p* = 0.38), with greater theta activity for incorrect than correct actions. Similarly, the ANOVA between on- and off-condition PD showed a significant main condition effect (*F*_(14,1)_ = 4.81, *p* = 0.0456; *η*^2^*p* = 0.26), with higher values for incorrect (M = −2.66) than correct (M = −16.12) actions. The ANOVA between HCs and off-condition PD also showed a significant main condition effect (*F*_(27,1)_ = 8.45, *p* = 0.0071; *η*^2^*p* = 0.24) and a significant condition vs group interaction (*F*_(27,1)_ = 6.31, *p* = 0.0182, *η*^2^*p* = 0.19). Post hoc analyses of this interaction showed a significant difference between correct and incorrect actions in the HCs only (*t*_(13)_ = −3.22, *p* = 0.031; M_COR_ = −14.82, M_ERR_ = 3.38), and an inter-group difference for incorrect actions (*t*_(26.13)_ = 2.58, *p* = 0.032; M_ERR_ [HCs] = 3.38, M_ERR_ [off-condition PD] = −16.78). However, there was no significant difference between conditions within off-condition PD (*t*_(14)_ = 0.44, *p* = 0.740; M_COR_ = −18.37, M_ERR_ = −16.78). Therefore, there was no theta-power increase with incorrect actions. The theta activity in electrode FCz is shown in Figs. 3 and 4.

The ANOVAs on the differentials showed no significant effects when comparing HCs and on-condition PD and on- and off-condition PD. However, there was a significant main group effect when comparing HCs and off-condition PD (*F*_(27,1)_ = 6.31, *p* = 0.0182; *η*^2^*p* = 0.19). Post hoc analyses of this main group effect showed that off-condition PD had lower theta power in the differential activity (M_DIFF_ = 1.59) than the HCs (M_DIFF_ = 18.20; *t*_(22.35)_ = 2.47, *p* = 0.021). Therefore, the increase in action error-related theta activity was lower in off-condition PD than in HCs.

The ANOVAs comparing the oPe (differential) and theta (differential) after z-score transformation on different electrodes (FCz/Pz) and response type (oPe/theta) showed no effects within or between groups. Similarly, to highlight potential differences between delta and theta-power activities, we also performed an ANOVA comparing theta (differential) and delta (differential) within each group and three ANOVAs between groups comparing both electrodes (FCz/Pz) and frequencies (delta/theta). The within-group analyses showed a significant difference between differential delta and theta only in the off-condition PD for the main frequency factor (*F*_(14,1)_ = 5.31, *p* = 0.0370; *η*^2^*p* = 0.28), with greater delta (M = 16.77) compared to theta (M = 1.59) power. No power difference between these frequencies was found between on-condition PD and HCs. The between-group analyses showed no significant differences in delta and theta activity.

The effect sizes (*η*^2^*p*) of differential theta activity show a large effect in the magnitude of between off-condition PD vs HCs (*p* = 0.0182; *η*^2^*p* = 0.19) and off- vs. on-condition PD (*p* = 0.1466; *η*^2^*p* = 0.14), while a small effect between on-condition PD vs HCs (*p* = 0.2544; *η*^2^*p* = 0.05). The effect sizes of the main response effect (oPe/theta) in the analysis that considered the differential of both oPe and theta after z-score transformation show a medium effect in the off-condition PD (*p* = 0.1895; *η*^2^*p* = 0.12), and a small effect in on-condition PD (*p* = 0.7726; *η*^2^*p* = 0.01) and HCs (*p* = 0.4213; *η*^2^*p* = 0.05). The effect sizes of the main frequency effect (delta/theta differential) show a large difference in magnitude in the off-condition PD (*p* = 0.0370; *η*^2^*p* = 0.28), while small effects have been found in the on-condition PD (*p* = 0.4459; *η*^2^*p* = 0.04) and HCs (*p* = 0.5100; *η*^2^*p* = 0.03).

### Other EEG frequencies potentially involved in error monitoring

#### Delta (2–4 Hz)

##### Cluster-based statistics

**Fig. 5:**
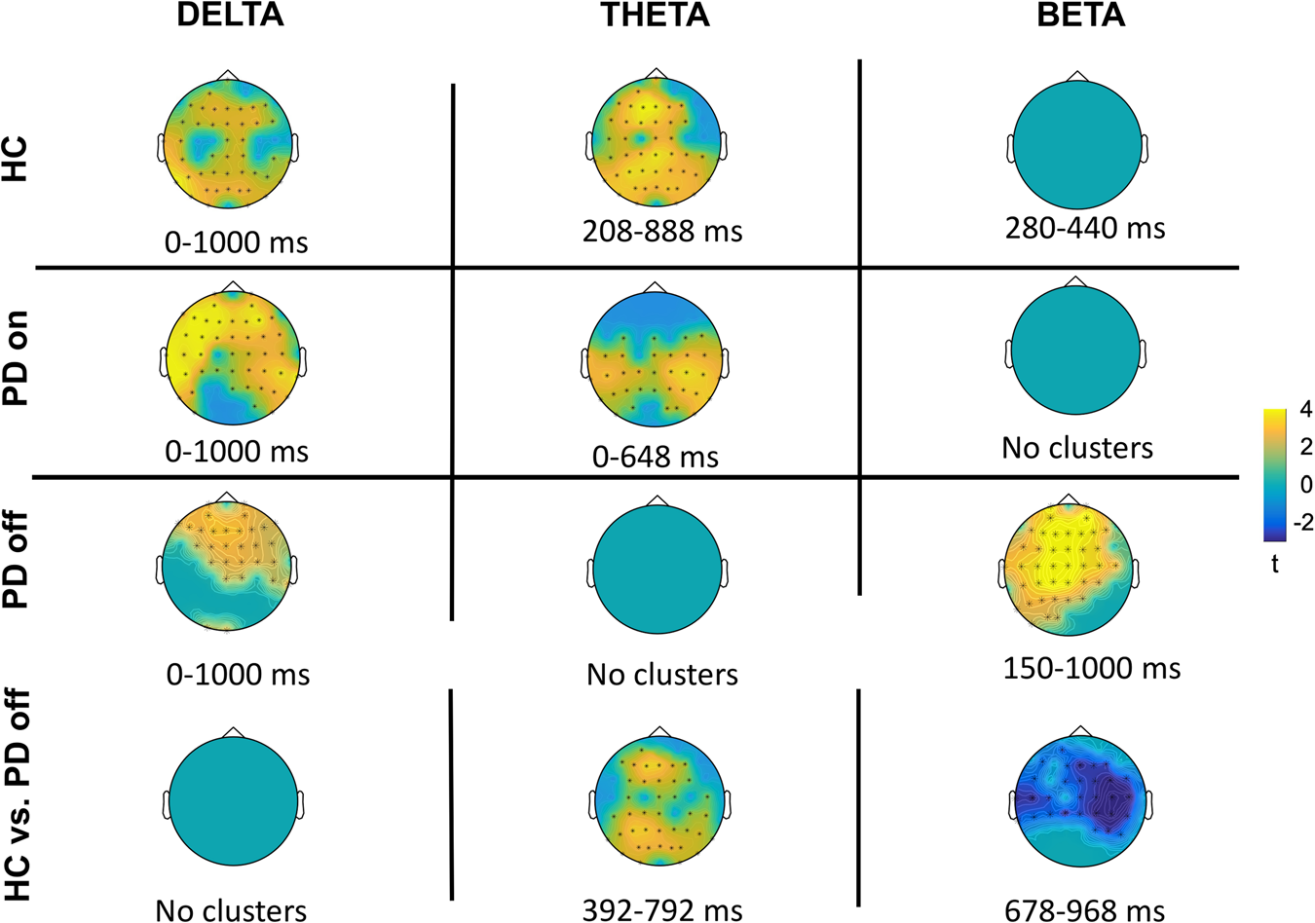
Cluster-based permutation in the TF domain for each group. Scalp representation of the cluster-based permutation (dependent sample *t*-test with cluster-correction at *p* < 0.05) of incorrect vs correct action. For each frequency and group, the topography shows the time-point at which the cluster reached the maximal spatial extension in the interval of interest. The intervals with a significant effect are shown below the topography. The bottom line shows cluster-based comparisons of the differential activity (incorrect minus correct) in the frequency bands of interest between HCs and off-condition PD; only intervals with significant clusters are shown.

We found significant differences within the HCs (*p* = 0.0080, range 0–1000 ms), on-condition PD (*p* = 0.0020, range 0–1000 ms), and off-condition PD (*p* = 0.0040, range 0–1000 ms) groups. In all three groups, clusters showed greater delta activity for incorrect than correct actions (Fig. 5). The difference was more prominent in the frontal and parietal areas in the HCs, but more prominent in the frontocentral areas in both groups of PD patients. There were no significant differences between groups when comparing the maps of incorrect and correct actions.

#### Alpha (8.1–12.3 Hz)

##### Cluster-based statistics

No significant alpha activity differences were associated with incorrect than correct actions in any group.

#### Beta (12.3–30.6 Hz)

##### Cluster-based statistics

Cluster-based permutation comparing incorrect and correct actions indicated a trend in the HCs (*p* = 0.0799, range 280–440 ms) with a central-contralateral distribution opposite to the observed arm (Fig. 5). On-condition PD showed no significant difference in condition. However, off-condition PD showed a significant difference (*p* = 0.0040, range 150–1000 ms), mainly in the central electrodes. The independent-sample comparison between groups indicated a significant difference between the HCs and off-condition PD (*p* = 0.0359, range 678–968 ms), reflecting higher beta power in the central areas with off-condition PD than with HCs (Fig. 5; see Discussion).

### Subjective reports during the virtual reality-EEG task

A lower sense of ownership was reported for incorrect than correct trials in all three groups (M ± SD; HC_COR_ = 58.09 ± 24.02 and HC_ERR_ = 57.22 ± 26.27; on-condition PD_COR_ = 65.37 ± 28.41 and PD_ERR_ = 63.77 ± 29.58; off-condition PD_COR_ = 59.60 ± 28.84 and PD_ERR_ = 58.75 ± 27.20). However, within- and between-group analyses showed no significant group or condition differences (all *p* > 0.05). In addition, no significant correlations were found. Overall, participants in the three groups responded correctly to the catch questions, confirming their engagement in the task and their understanding of the observed action (correct answers: HCs = 94%, on-condition PD = 97%, and off-condition PD = 93%).

## Discussion

To explore dopamine’s influence on the electrocortical dynamics of error monitoring, we recorded EEG in patients with PD as they observed correct and incorrect actions performed by a virtual arm observed from a 1PP. We used a within-subject approach to test the same PD patients under dopaminergic medication (on-condition) and after dopaminergic withdrawal (off-condition). A control group of healthy participants was also included.

Our first observation was that theta power increased contingent upon observation of incorrect actions in HCs and on-condition PD patients but not in off-condition PD patients, indicating that dopamine depletion modifies this neurophysiological performance monitoring marker. Our second observation was that, unlike theta activity, oPe and delta power were higher for incorrect than correct actions in HCs and on- and off-condition PD patients, indicating that error monitoring comprises processes that may or may not be impacted by levels of dopamine balance. Furthermore, off-condition PD patients showed a stronger error-related beta response with incorrect than correct actions, also when comparing the differential activity of off-condition PD with HCs. No such effect was observed with on-condition PD patients.

In response to incorrect actions, off-condition PD patients showed abnormal theta-band activity with no power increase. Crucially, when the same PD patients were tested just after taking their regular dopaminergic medication (on-condition), theta activity in response to incorrect actions was restored, leading to the same pattern as HCs^22^. It should be noted that consistent with previous studies^63,64^, we did not observe a difference in theta activity contingent upon error monitoring between on- and off-condition PD. The fact that PD patient responses did not differ between their on- and off-conditions might reflect different factors, including variability in individual responses to dopaminergic treatment^16^ and dopaminergic neuron degeneration in their VTAs^67^. Importantly, a significant theta-power difference in response to incorrect actions was found between HCs and off-condition PD patients. Our off-condition PD patients had a longer withdrawal phase (~18 h) and increased extrapyramidal motor symptom severity (mean UPDRS = 37) from their dopaminergic medication compared to previous studies^11^. These factors might have allowed us to better highlight dopamine’s contribution to performance monitoring compared to previous studies using a shorter withdrawal period that found no differences^36,64^. Tellingly, no differential error-related theta activity was found between HCs and on-condition PD patients. Also, despite not showing a significant difference between oPe and theta, off-condition PD qualitatively showed a medium effect size, that was greater than the two other groups. In addition, to highlight potential differences between error-monitoring processes, analyses on theta and delta differentials showed a significant difference and a large effect size in the response type in off-condition PD patients (i.e. an increased delta—but not theta—power); this effect was not shown by the other two groups. Delta and theta differential activities, differently than oPe (ERP) and theta (TF), may be more informative to compare as they both consider also EEG activity which is not time- and phase- locked with the event. This result further suggests dopamine’s central role in performance monitoring related to mid-frontal theta and in regulating information precision during predictive processes triggered by salient and unexpected events^18^, as recently reported in the social context^68–70^. Previous studies suggested a link between fronto-parietal theta activity and executive functions, such as working memory^71^ and eye-blink decay^72^, which have been associated with executive dysfunction in PD. Others have found mixed effects such as beneficial or detrimental effects of the dopaminergic medication on the executive domain^73^. In this case, no significant correlation survived multiple comparison correction, suggesting a non-robust association between theta activity during error observation and behavioural indices of executive functions in the PD sample of this study.

Our results showed that observing incorrect actions produced detectable oPe in all groups. Both single-electrode and cluster-based permutation analyses showed that observing incorrect actions was characterised by increased ERP amplitude compared to observing correct actions. There was a topographical oPe centroparietal distribution and greater effects over the central (FCz) than the parietal (Pz) electrode. The spread of activity over the central rather than posterior electrodes is potentially consistent with studies showing that oPe is a cortical response characterised by subcomponents spreading over frontocentral and centroparietal electrodes^41,74^, that ageing and pathological populations often show component shifts from posterior to frontal areas, and that Pe/oPe are characterised by large topographical distribution variability across studies and clinical populations^41^.

While on-condition PD patients showed generally lower amplitude values than HCs, the differential waves (incorrect minus correct) showed no oPe response differences between groups. Notably, the non-significant difference in the oPe response between groups is consistent with the hypothesis that this component’s generation is independent of the dopaminergic system^59^. This evidence is consistent with findings in other clinical populations, such as patients with ACC lesions^8,9,75^ in which the Pe but not the ERN or theta was unaltered. Patients with lateral prefrontal lesions extending into the insula had reduced Pe responses^2,76^. This finding suggests that activities associated with Pe activity might engage a neural network involving the ACC and anterior insula and somatosensory areas^49^ that might be spared in PD.

Surprisingly, we did not find any oERN, an error detection marker present in previous studies with young adults^30,31,58^. However, it should be noted that this component was absent in older populations investigated with a paradigm very similar to ours^77^. We speculate that its absence may reflect weak oERN modulation in aged individuals^78–80^. Another related explanation is that TF analyses might be more sensitive to phase- and non-phase-locked activity during continuous actions (e.g. theta), while ERPs are more sensitive to discrete events (e.g. ERN;^81,82^). Indeed, previous data suggest that ERN is dominated by intermittent theta-band phase-locking^83^. However, observing an error also elicits non-phase-locked activity. Therefore, not all mid-frontal activity is associated with oERN generation^29,58^.

We also hypothesise that oERN’s absence might not be related to the missing embodiment rating effects because participants in the three groups still reported a general feeling of ownership over the virtual arm, albeit with non-significant differences across conditions^58^. Moreover, even when an oERN was not identified, the typical theta power increase in response to incorrect actions was observed. It should be noted that drawing firm conclusions on this point is complicated by sample characteristics and task novelty compared to traditional experimental setups. Moreover, oERN’s absence was somewhat unexpected. Future studies with ad hoc experimental designs are required on this important issue (e.g. investigating error responses across ages in different experimental setups).

Studies have shown that alpha^84^, delta^47,56^, and beta^85^ frequencies may potentially be associated with error-monitoring processes. In this study, no error-related modulation was found for the alpha band. Instead, delta activity was higher for incorrect than correct actions in all groups. This finding is consistent with the belief that in a filtered signal, delta activity is associated with the Pe/oPe response in the time domain^47,56^. Interestingly, our results showed how this error-monitoring marker is not influenced by dopamine depletion. The enhancement in error-related delta activity and oPe in response to observing incorrect actions in HCs and on- and off-condition PD suggests that this mechanism is preserved in PD. Moreover, this finding contrasts with the absence of an error-related theta-response in off-condition PD patients, suggesting that dopaminergic projections may not have a prominent direct role in error-related delta and oPe generation.

Analysis of beta-band showed error-related increased in PD off, within-group and also when contrasted with HCs. Beta rhythm has been associated to sensorimotor control^86–88^, learning tasks^89^, and long-distance communication between visual and sensorimotor areas^90^. Previous studies on epileptic patients showed that alpha and beta frequency modulation was present while executing and observing reach and grasp actions^91^. Moreover, lower rhythms, such as delta and theta (<8 Hz), were more involved during movement execution than observation^92^. These results suggest that parallel neurophysiological processes (e.g. beta frequencies) can be observed during different movement modalities. Local subthalamic nucleus field recordings have associated excessive beta activity in PD patients with pathophysiological motor symptoms^93^ that were restored by dopaminergic treatment^94,95^. Previous studies indicate that beta rebound was stronger after incorrect actions, suggesting beta’s potential role in evaluating action significance and active response inhibition^85^. Off-condition PD patients showed a stronger error-related beta response in this study. However, no such effect was observed in on-condition PD patients in whom dopaminergic medication appears to suppress beta activity^94^. Therefore, while HCs showed greater theta than beta involvement in error responses, off-condition PD patients appear to show the opposite pattern. Future studies must investigate whether PD compensates for the involvement of mid-frontal theta with higher frequencies during dopaminergic withdrawal. It may be of particular interest to explore whether increased beta activity might be detrimental^57^ or represent a compensatory mechanism rather than a pathophysiological marker^96^.

Concerning the relation between the UPDRS-H&Y and EEG signals, no significant correlation was found. Similar lack of correlation was found also in previous reports^55^, in which patients in on-condition had lower scores than off-condition in the UPDRS-III, but this result did not correlate with the EEG signal. Further, the scores obtained with the UPDRS III are related to patients ‘motor ability’ and it allows to evaluate the efficacy of the dopaminergic medication in improving general motor symptoms; however, this might be not directly related with the error-monitoring signals obtained within this task, in which a direct and active motor performance was not required.

Even when participants qualitatively reported a greater sense of ownership during the performance of a correct rather than incorrect action, analyses of the embodiment ratings did not show a significant difference between conditions. This result contrasts with previous studies on young adults^30,31,58^. While somewhat speculative, several explanations for between-study differences can be proposed. First, we tested old adults in this study, contrasting with our previous reports. Second, we asked participants to report embodiment over the artificial limb in 30% rather than 100%^30^ of trials and only asked questions about feelings of ownership and not agency, which has also been associated with action monitoring^96–98^, to avoid long sessions and prevent patient fatigue. However, despite not showing a significant difference between incorrect and correct actions, our participants reported a general feeling of ownership that was >50% on the 0–100 VAS scale, consistent with previous reports on the feelings of embodying a virtual arm^31,58,99^. Therefore, we suggest that participants feel ownership of their virtual arm and that the process we are studying can be defined as monitoring actions performed by an embodied virtual arm.

Previous studies have highlighted the advantages of using a virtual task to induce the illusion of committing an error from a 1PP^30^. Here, correct and incorrect actions are performed by an arm calibrated to the participants’ body size, something that would be impossible with traditional setups. Therefore, many recent studies have suggested that virtual reality could be a promising technique for cognitive and motor rehabilitation^100^. PD patients mostly answered the catch questions correctly. Indeed, we expected a high accuracy rate since the assignment was extremely easy and not performed under time-pressure conditions.

Our approach allowed us to directly investigate how distinct electrocortical signatures to errors are differently affected by dopamine balance in PD patients. This study’s strengths include using an ecological and short (~20 min) immersive virtual reality task that tested the brain’s reaction to errors in PD patients without confounding effects due to movement speed or difficulty^101^. Participants qualitatively reported a greater sense of ownership during correct rather than incorrect actions. However, while their scores were consistent with previous studies^30,31^, statistical analyses did not show significant differences between conditions.

We acknowledge some potential limitations. First, PD patients were tested twice, while HCs were tested only once. However, consistent with previous studies^55^, we can reasonably exclude a learning or habituation effect because the adopted task involved simple action observations and did not require the acquisition of task-specific abilities. Moreover, reactivity to errors is due to their lower frequency, making them odd events. Crucially, we have already shown that electrocortical error detection and monitoring markers are found even when incorrect events comprise 70% of trials^30^. This finding suggests that the deviation from expected motor plan executions is perceived as a salient and significant event independent of its occurrence frequency^30^. Therefore, the brain’s reaction to the observed motor errors found in this study cannot be ascribed to an oddball effect^79^. Second, we did not record the electromyography to monitor real movements during the experiment, although we asked participants to remain still and avoid movement. Third, the main analyses were performed using two electrodes (FCz and Pz) that were specified a priori based on previous studies. However, this could have hidden the signal’s richness. To compensate we also provided all-brain electrode analyses but approaches other than cluster-based permutation to identify electrode clusters in a button-up manner could be relevant for investigating the involvement of single or multiple brain regions. In particular, studies with intracranial electrodes^102^ may have allowed us to circunvent the electrode level limitations due to volume conduction issues and identify cortical regions involved in specific monitoring processes.

Finally, other neurotransmitters might play a role in performance monitoring through direct ACC modulation or their influence on the dopamine system^55,103^. Future studies should explore the role of neurotransmitters, such as serotonin, norepinephrine, gamma-aminobutyric acid (GABA), and adenosine in cooperation with dopamine in orchestrating error processes^33^ in cohorts with different phenotypes^104^.

In conclusion, this study expanded research on the complex architecture of the monitoring system in older adults and neurological populations by providing support for the hypothesis that error-related theta activity is influenced by dopamine balance^44,48,105^. Error-related theta activity modulation contingent upon dopamine depletion found in this study may provide a foundation for future studies on neurophysiological biomarkers related to prediction processing and model updating^38,106,107^ that may ultimately help to understand error monitoring during action observation in PD patients.

## Methods

### Participants

Seventeen patients with PD participated in this study. The MorePower (version 6.0.4^108^) software was used to compute the sample size. It indicated that 14 participants would be sufficient to provide 85% power with an alpha of 0.05 and a partial η^2^ of 0.4 based on a previous study using the same paradigm to assess the electroencephalographic error-monitoring markers^58^. All participants had normal or corrected-to-normal visual acuity. The inclusion criteria were: (i) idiopathic PD diagnosis according to the United Kingdom PD Society brain bank criteria on the unified PD rating scale (UPDRS^109^); (ii) a mini-mental state examination (MMSE) score >26^110^; (iii) the absence of other neurological and psychiatric diseases; (iv) treatment with daily doses of dopamine or dopamine agonists (equivalent levodopa doses; see Table 2 for more details). One patient was excluded due to possible incorrect medication and a lack of motor scale data. One patient withdrew from the study. Therefore, our final group of 15 PD patients comprised five females and ten males aged (mean [M] ± standard deviation [SD]) 70 ± 9 with 12 ± 4 years of education.

**Table 2:** Patients’ demographic, clinical and a subset of neuropsychological tests which tap executive functions.

Subject	Age	Education	Illness duration (months)	L_Dopa equivalent	BDI	MMSE	MMPSE	TMT_A	TMT_B	TMTB-A	WCSTcat	WCST_p	WCST_np	MMSE_pc	MMPSE_pc	TMT_A_pc	TMT_B_pc	TMTB-A_pc	WCSTcat_pc	WCST_p_pc	WCST_np_pc
P01	83	8	264	870	0	29	31	61	131	70	6	1	0	28.7	32.57	29.05	26.29	0	6	0	0
P02	58	13	108	650	11	29	31	30	72	42	6	2	1	26.2	30.07	13.95	16.44	2.47	6	1.83	0.5
P03	77	13	84	750	5	29	28	51	99	48	6	1	0	27.3	28.38	30.23	37.48	7.21	6	0	0
P04	58	8	276	810	12	28	31	110	265	155	6	0	3	26	30.94	94.1	209.91	115.79	6	0	2.51
P05	72	13	156	600	4	29	29	36	133	97	6	0	0	26.7	29.05	19	83.11	64.09	6	0	0
P06	70	13	84	550	5	30	31	51	117	66	6	0	1	30	31.05	34.49	68.65	34.13	6	0	0.48
P07	78	5	204	400	13	29	25	86	326	240	4	5	9	28.7	26.77	52.78	212.17	159.36	4.32	3.05	7.56
P08	82	8	24	3125	0	29	25	55	127	72	4	3	13	28.7	26.57	23.77	24.5	0.69	4.38	0.49	11.79
P09	72	13	30	425	3	30	30	46	81	35	3	3	12	30	30.05	9.62	0	0	3.21	1.83	10.76
P10	60	13	84	725	7	30	31	20	77	57	4	5	15	30	30.4	10.27	49.6	39.31	4.06	4.75	14.79
P11	68	5	24	750	21	29	32	61	246	185	5	3	8	27.9	32	34.14	151.84	117.68	5.17	2.16	6.86
P12	57	18	36	400	6	28	31	19	74	55	4	6	10	25.2	29.38	18.38	83.4	65	4.04	5.89	10.22
P13	79	13	288	650	7	30	NA	55	137	82	6	1	7	30	NA	33.27	72.51	39.2	6	0	6.23
P14	68	18	144	950	18	29	32	30	83	53	3	4	8	26.2	32	22.1	69.89	47.76	3.17	3.15	7.88
P15	64	13	156	650	5	29	30	43	104	61	6	0	1	26.2	29.4	31.53	102.14	70.58	6	0	0.67
														cut-off:							
														23.8	22.85	94	283	187	4.25	7.65	10.75

PD Patients were tested during on-condition. The column labelled “L-Dopa equivalent” reports the daily dose of dopamine or a dopamine agonist taken by each patient. On the left side of the table demographic and clinical data are shown; in the central section of the table, raw scores for each patient are reported, while on the right-side of the table, the corrected values are shown (“pc” stands for post correction for age-education). Cut-off scores for each neuropsychological test are also reported at the end of the table. *MMSE* mini-mental state examination, *MMPSE* Mini-mental Parkinson State Examination, *BDI* Beck depression inventory, *TMT_A* trial-making test A, *TMT_B* trial-making test B, *TMT_B-A* trial-making test BA, *WCST_CAT* Wisconsin Card Sorting Test_categories, *WCST_P* Wisconsin Card Sorting Test_perseverative errors, *WCST_NP* Wisconsin Card Sorting Test_non perseverative errors.

Sixteen healthy participants served as HCs. One HC was excluded due to impaired vision, and one due to MMSE below cut-off. Therefore, our final group comprised 14 HCs matched for age and education with the PD patients: five females and nine males aged 70 ± 6 with 13 ± 3 years of education. HCs were included according to the following criteria: (i) absence of neurological or psychiatric diseases in anamnesis; (ii) absence of subjective cognitive disorders; (iii) absence of medications with psychotropic action; (iv) MMSE score >26^110^(see Table 1a for details).

PD patients received an extensive neuropsychological assessment during their on-condition (under treatment), as part of a specialised hospital clinical practice. Demographic and clinicals information of PD patients and main neuropsychological tests that have been considered of interest for the current study are shown in Table 2.

All participants were naïve to the purposes of this study and provided written informed consent. The experimental protocol was approved by the local Ethics Committee at the IRCCS Santa Lucia Foundation of Rome (reference number: CE/PROG.533) and performed according to the ethical standards in the 2013 Declaration of Helsinki.

### Apparatus and stimuli

**Fig. 6:**
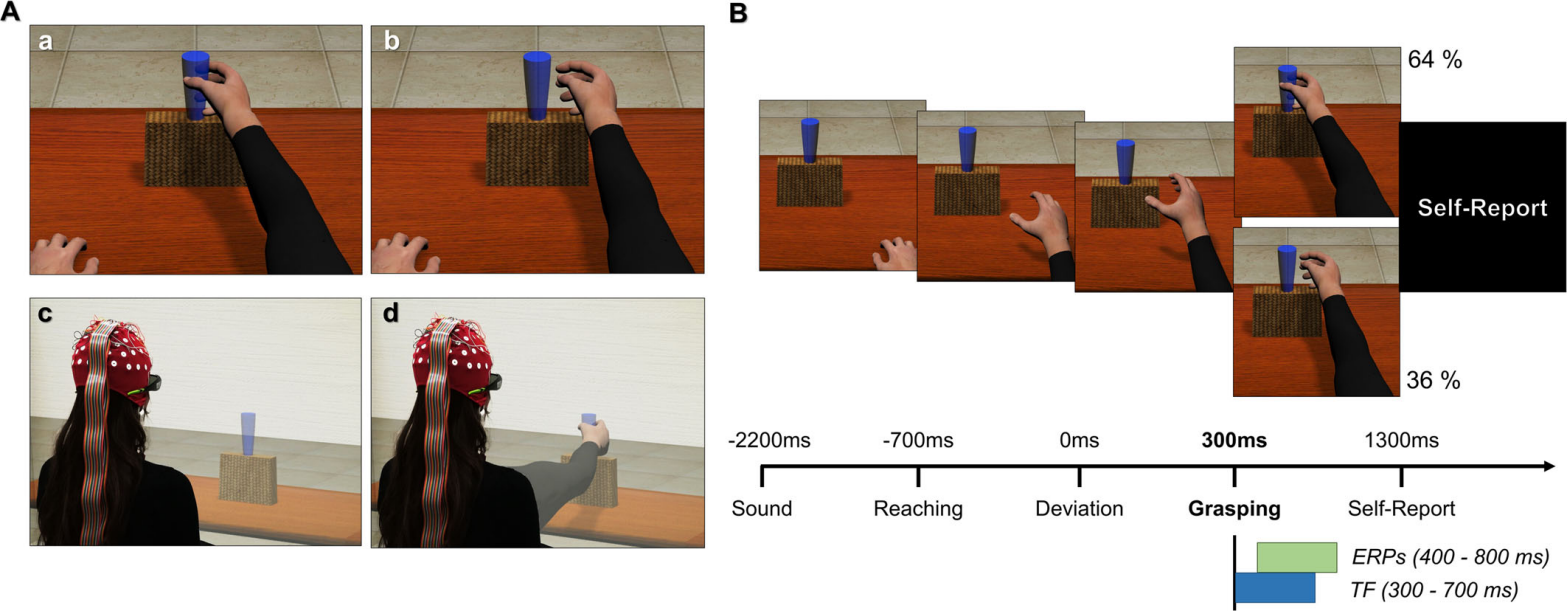
Schematic representation of the experimental paradigm and setup. **A** Images (c) and (d) show the participant immersed in a virtual scenario in the CAVE system while observing the real-size virtual arm from a 1PP during a correct (a) or erroneous (b) grasping action. **B** The timeline of a single trial. The avatar’s action lasted ~1000 ms, with the reaching phase equal for both outcomes. The onset of the avatar’s arm-path deviation is at 0 ms, and the end of the avatar’s action occurs at 300 ms. The time windows for event-related potential (ERPs) and time-frequency (TF) analyses have been chosen a priori based on previous studies^30,58^.

Participants sat in a Cave Automatic Virtual Environment (CAVE) with projectors directed to four walls of a room-sized cube (3 × 3 × 2.5 m^111^). The virtual scenario consisted of a basic room with a Table (1:1 scale). A dark yellow parallelepipedon was located in the centre of the table with a blue glass on top of it. The virtual glass was placed in the participant’s peripersonal space at a distance of ~50 cm. Participants observed a virtual right arm projected outside their right shoulder and congruent in dimension and shape with their body from a 1PP (see Fig. 6, panel A). The virtual arm and the scenarios were created using Autodesk Maya 2015 and 3DS Max 2015, respectively. The kinematics of the avatar were realised in 3DS Max and implemented in the virtual scenario as an animated 3D mesh. The virtual reality-EEG experiment used a 3D immersive virtual environment rendered in CAVE using XVR 2.1^112^. Participants observed the virtual environment displayed through Optoma 3D active glasses. Their head position was tracked in real-time by an Optitrack System comprising eight infrared cameras placed inside the CAVE.

### Experimental procedure

Patients were tested using different sessions on separate days. First, an extensive neuropsychological assessment was made while patients were under their dopaminergic treatment to ascertain their cognitive profile as part of standard clinical practice. For the experimental virtual reality-EEG task, the patients visited the laboratory in two separate sessions 15 days apart. In one session, they were examined within 60 min of their first medication intake (on-condition). In the other, they were examined after an 18 h washout from their dopaminergic medications used to treat PD (off-condition^113^). The order of on-off-condition examinations was balanced across participants.

Before the virtual reality-EEG experiment began, participants underwent a calibration phase where the size and the position of the virtual right arm were adapted to their real arm. A short resting-state period was included before the experiment, which was not of interest to this study. Next, they performed a brief practice session (eight trials, four correct, and four erroneous) in which they familiarised themselves with the virtual arm’s movements and the task. Each participant was requested to passively observe the virtual arm’s movements (avoiding any movements of their real arm) and was informed that the goal of the movements was to reach and grasp the glass on the table. They were also informed that the action might or might not be successful.

The virtual reality-EEG task consisted of 110 trials per participant (70 correct and 40 incorrect virtual arm movements) with a total duration of ~20 min. Including a smaller proportion of erroneous to correct actions was consistent with previous studies in which errors are often a salient infrequent event^47^. Previous studies using the current paradigm have used 30%:70% incorrect-correct actions^30,31^ and their inversion (70%:30%^58^). In this study, the choice to include a smaller number of erroneous (36%) to correct (64%) actions was preferred to maintain the infrequency of errors and provide a sufficient number of trials in this clinically aged population.

At the onset of each trial, a sound signalled the beginning of the action. During the trial, participants passively observed the movement of the virtual right arm from a 1PP. The total duration of the movement was 1000 ms. The movement’s kinematics were identical for 70% of the action duration in both correct and incorrect conditions but could diverge in the final 30% leading to either a successful or unsuccessful grasping;^30,31,58,77^ here a short videoclip of the paradigm: https://osf.io/rxeha). The deviation from the to-be-grasped object was identical in all erroneous trials (Fig. 6, panel B). The sequence of correct and incorrect trials was pseudorandomised. After the end of the action, the avatar’s arm remained still for 1000 ± 50 ms before the screen went black.

During the inter-trial interval (ITI), one of three events occurred: (i) participants had to answer a catch question ‘did the arm grasp the glass?’ (yes/no) to confirm their engagement in the virtual reality-EEG task in 10 out of the 110 trials (4 incorrect and 6 correct); (ii) an empty black screen was presented in 65 out of the 110 trials; (iii) participants had to rate their illusory sense of embodiment over the virtual arm in 35 out of the 110 trials (13 incorrect and 22 correct). The illusion was verbally rated on a visual analogue scale (VAS) between 0 and 100 by answering the question, ‘to what extent did you feel the virtual arm was yours?’ (0 = no ownership, 100 = maximal ownership^114–118^). In the first and third event types, the black screen lasted until a vocal response was given. In the second event, the experimenter pressed a key to start the next trial, producing a variable ITI (M duration = ~4.000 ms).

After each EEG session, an expert neurologist administered the UPDRS-III to PD patients^109^—a 27-item scale in which each item is evaluated on a 5-point Likert scale (from 0 to 4), where the maximum total score of 108 reflects the highest disability level—and the Hoehn and Yahr scale^119^. These scales estimate the patients’ motor performance and disease progress, respectively, and allowed us to evaluate the efficacy of dopaminergic medication in improving motor symptoms. Higher scores indicated higher disease severity, with levels <2 on the UPDRS-III found in healthy elderly^120^. The two scales were administered under both on- and off-condition PD patients.

### EEG recording and processing

EEG signals were recorded using a Neuroscan SynAmps RT amplifier system and 62 scalp electrodes embedded in a fabric cap (Electro-Cap International), arranged according to the international 10–10 system. Horizontal electrooculogram was recorded bipolarly from electrodes placed on the outer canthi of each eye. Online EEG signals were recorded continuously in alternating current mode with a bandpass filter (0.05–200 Hz) and a sampling rate of 1000 Hz. Impedances were kept below 5 kΩ. All electrodes were physically referenced to an electrode placed on the right earlobe and re-referenced offline to the common average across all electrodes. Offline, raw data were bandpass filtered with a 0.1–100 Hz filter (finite impulse response filter, transition at 40–42 Hz, and stopband attenuation at 60 dB). Independent component analyses (ICA^121^) were performed on the continuous EEG signal, and components that were clearly related to blinks and ocular artefacts were removed (5.8 ICA components on average).

Event-related potential (ERP) analyses used an additional bandpass filter (0.3–30 Hz) applied to the continuous raw signal. The EEG signal was then downsampled to 500 Hz and epoched in wide windows 3 s in length, from −1.5 to +1.5 s, to avoid edge artefacts induced by the wavelet convolution in the TF analysis. Epochs were time-locked (0 ms) at the avatar’s arm-path deviation, with direct current offset correction to the previous 300 ms preceding the deviation^29,58^. Each epoch was then visually inspected to remove residual artefacts (e.g. eye blinks) by checking for epochs exceeding ±100 µV amplitude^122^. After this procedure, a small proportion of trials were removed from the original datasets (HCs: 4.5%; on-condition PD: 1%; off-condition PD: 4%). Therefore, each group had a sufficient and comparable number of trials for correct (COR) and incorrect (INC) actions (M ± SD; HC_TOTAL_: 105 ± 5, HC_COR_ = 67 ± 3, and HC_INC_ = 38 ± 2; on-condition PD-ON_TOTAL_: 109 ± 3, PD ON_COR_ = 69 ± 4, and PD ON_INC_ = 39 ± 1; off-condition PD-OFF_TOTAL_: 106 ± 5, PD OFF_COR_ = 67 ± 3, and PD OFF_INC_ = 39 ± 2^123^).

Unless otherwise specified, data were normally distributed (Shapiro–Wilk test), and parametric analyses were used. EEG analyses were performed using the Brainstorm toolbox (https://neuroimage.usc.edu/brainstorm/^124^) and custom Matlab routines. Statistical analyses were performed using R software^125^. Effect sizes were calculated using Cohen’s *d*. ERPs and TF statistical analyses were performed using the *erpR* R package^126^. Analyses excluded practice trials.

### EEG analyses

#### Time-domain analysis

All ERP analyses were based on mean amplitude, as recommended by Luck and colleagues^127^. We analysed ERPs at a whole brain level using a time-point cluster-based permutation analysis with 1000 repetitions (using *p* < 0.05 to identify significant cluster differences), Monte-Carlo correction in the 0–1000 ms time window on all electrodes, and cluster comparisons within and between groups to assess neural pattern reliability and robustness over neighbouring data points^128,129^. In addition, analyses using all electrodes were used to capture potential data-driven modulations at the all-electrode level, which can be relevant in ageing and clinical populations where shifts in neural activation might be observed^130^. Moreover, traditional ERP analyses were performed. We did not analyse oERN since it was not observed during visual inspection of the time series in the a priori selected time window (see Discussion). The oPe is a P300-like component maximally peaking at electrode Pz distributed on the frontal electrodes (e.g. FCz^41^).

Separate analyses of variance (ANOVAs) were computed on a priori established time windows of interest (400–800 ms) to be consistent with previous studies^30,31^. Since PD patients have been tested twice (within factor) and HCs have been tested only once, three separate ANOVAs were computed: HCs vs on-condition PD including two within-subject factors (condition: inc or cor; electrode: FCz or Pz) and one between-subject factor (group: HCs or on-condition PD); on- vs off-condition PD including three within-subject factors (condition: inc or cor; group: on or off; electrode: FCz or Pz); HCs vs off-condition PD including two within-subject factors (condition: inc or cor; electrode: FCz or Pz) and one between-subject factor (group: HCs or off-condition PD). Comparisons with significant effects were subject to post hoc analyses with false discovery rate (FDR) corrections for multiple testing.

In addition, following golden-standard recommendations^127,131^, the ERP differential obtained by subtracting the correct from the erroneous condition was compared using three ANOVAs: on- vs off-condition PD, HCs vs on-condition PD, and HCs vs off-condition PD including two factors (group: HCs, on-condition PD, or off-condition PD; electrode: FCz or Pz). Comparisons with significant effects were subjected to post hoc analyses with FDR correction for multiple testing.

We also directly compared the z-transformed values of the oPe and theta power’s differential activities to identify potential differences among the three groups, although these indices are obtained with different mathematical transformations and indices from the TF domain include also non-time and phase-locked activity. To this aim three ANOVAs considering electrode (FCz and Pz) and response (oPe and theta) were performed as well as three ANOVAs between groups; we could not perform one unique ANOVA in order to respect the dependency among the observations (i.e. PD are the same individuals). However, significance testing only allows rejecting the null hypothesis; it does not disentangle whether the null hypothesis should be accepted, or the lack of effect is due to inconclusive data^132^. Therefore, we examined evidence favouring the null within (on- and off-condition PD) and between (on- and off-condition PD vs HCs) group effects on oPe. We performed a Bayesian paired sample *t*-test comparing oPe’s differential index (incorrect minus correct) between on- and off-condition PD groups. We also performed two separate Bayesian independent-sample *t*-tests comparing oPe’s differential index between HCs and on- or off-condition PD. This approach enabled us to determine whether the absence of a significant group effect could be interpreted as evidence favouring the null effect of the group on oPe.

Bayes factors (BFs) were computed using the Jasp statistical software Jasp (https://jasp-stats.org/^133^) with default uninformative priors (Cauchy scale = 0.707). BF_10_ values >3 and >10 indicate moderate and strong support for the alternative hypothesis, respectively. BF_10_ values <0.1 and <0.33 indicate strong and substantial support for the null hypothesis. BF_10_ values 0.33 < BF10 < 2 are inconclusive.

#### TF domain analysis

We used a complex Morlet transformation to compute TF decomposition in the TF analysis. A mother wavelet with a central frequency of 1 Hz and time resolution of 3 s (full width half maximum) was designed in the Brainstorm software^124^. The other wavelets were computed from this mother wavelet and ranged from 1 to 80 Hz in 0.5 Hz logarithmic frequency steps. We separately normalised each signal and frequency bin relative to the baseline by computing the relative power change (%) over the TF decomposition as ERSP*(f,t)* = S*(f,t)* − S_base_ /S_base_, where S(t, f) is the signal spectrum in a specific interval of time (t) and frequency (f), and S_base_ represents the reference signal’s mean power in the baseline interval (event-related spectral perturbation [ERSP]). To avoid edge effects, the power activity from −700 to −500 ms—the window in which the avatar’s movement was identical in erroneous and correct conditions—was used as the baseline interval (S_base_). Positive and negative values indicate a decrease or increase in synchrony of the recorded neuronal population, respectively^87^, relative to a given reference interval when equal neural activity is expected. Here, a relative power increase or decrease represents a power modulation compared to the mean power activity at baseline.

We investigated the effect on the whole brain using time-point cluster-based permutation analyses with 1000 repetitions for each run (*p* < 0.05) and Monte-Carlo correction on a wide time window of 0–1000 ms to visualise the distribution on the scalp^55^^,134^. Cluster comparisons within and between groups were performed. In addition, consistent with previous studies^29–31,58^, the main theta activity analyses were computed focusing on the theta band (4–8.1 Hz) in the preselected time interval (300–700 ms) corresponding to 400 ms from the end of avatar’s action.

Besides frontal regions, the brain’s posterior regions were engaged in observational tasks involving passive observation of reaching movements^135^. In addition, an occipitotemporal theta-band increase was observed during the processing of body-part stimuli^29,136^. Similar to the ERP analyses, the theta analyses involved three separate ANOVAs among groups: one comparing HCs to on-condition PD ‘including two within-subject factors (condition: inc or cor; electrode: FCz or Pz) and one between-subject factor (group: HCs or on-condition PD); one comparing on- and off-condition PD including three within-subject factors (condition: inc or cor; group: on or off; electrode: FCz or Pz); and one comparing HCs and off-condition PD including two within-subject factors (condition: inc or cor; electrode: FCz or Pz) and one between-subject factor (group: HCs or off-condition PD).

Next, similar to the ERP analyses, the theta band’s differential index (incorrect minus correct^137^;) was compared. Comparisons with significant effects were subject to post hoc analyses with FDR correction for multiple testing. Besides theta (4–8.1 Hz), cluster-level analyses were performed on potential frequencies interest for error-monitoring processes in PD:^56,57,85^ delta (2–4 Hz), alpha (8.1–12.3 Hz), and beta (12.3–30.6 Hz). We were particularly interested in potential differences between delta and theta-power activities since they have been previously associated with distinct error-monitoring processes^56,138^ in the TF spectrum. Therefore, we also performed an ANOVA comparing theta (differential) and delta (differential) in each group and three ANOVAs comparing groups separately for both electrodes (FCz and Pz) and frequencies (delta and theta).

### Clinical and neuropsychological testing

Clinical data on motor ability relative to dopaminergic medication were analysed. We performed two ANOVAs with dopaminergic medication as a factor (on or off) separately for the UPDRS III and H&Y scales. Correlations between them and EEG signals were performed to investigate clinical deficits relative to EEG states during different dopaminergic conditions. Neuropsychological tests assessing executive functions and cognitive control were also taken into consideration in on-condition PD patients since previous data suggested a relationship between theta and ERN activity and performance in tasks underlying executive functions^32,60,71^. These included the trail-making test (TMT, parts B and BA^139^) and the Wisconsin card sorting test^62,63^. To assess the general cognitive functioning also the Mini Mental State Examination (MMSE^140^) and Mini Mental Parkinson State Examination (MMPSE^141^) were administered. We performed correlations between all-brain cluster-based permutation across time (0–1000 ms) in theta activity and the executive functions tests, with Montecarlo correction for multiple comparison, to observe the scalp distribution across electrodes. We also corrected the correlations for multiple comparison with false discovery rate (FDR) correction. In order to explore the link between clinical scales measuring patient’s motor disability and EEG states during motor action observation, we correlated the UPDRS and H&Y scales as measured in “on” and “off” and the EEG correlates (oPe, theta).

### Subjective reports during the virtual reality-EEG task

Embodiment ratings and the catch answers were calculated for correct and erroneous actions in the three groups as previously described^31,58^. The embodiment question, ‘How much did you feel that the arm was yours’ (on a 0–100 scale), was present only in a subset of trials (12% of incorrect and 20% of correct trials). Mean embodiment ratings for each trial type were calculated for each participant. The scores were compared with three separate ANOVA, including condition (correct or incorrect) as a factor, compared HCs to on- or off-condiction PD and on- and off-condition PD. We explored associations between the sense of embodiment and electrocortical error processing indices using Spearman’s rank correlations between embodiment ratings and theta error signatures. Each group’s percentage accuracy was calculated for the catch trials.

Statistical analyses of clinical-neuropsychological data and subjective reports were performed using the R software^125^. We applied the Greenhouse–Geisser non-sphericity correction and Bonferroni multiple testing correction when appropriate.

## Data Availability

The data are available in the Open Science Framework repository https://osf.io/z9rbu/.

## References

[1] Parkinson, J. *An Essay on The Shaking Palsy* (Sherwood, Neeley & Jones, 1817).

[2] Ullsperger M, Von Cramon DY (2006). The role of intact frontostriatal circuits in error processing. J. Cogn. Neurosci..

[3] Wylie SA (2010). Subthalamic nucleus stimulation influences expression and suppression of impulsive behaviour in Parkinson’s disease. Brain.

[4] Zavala B (2018). Cognitive control involves theta power within trials and beta power across trials in the prefrontal-subthalamic network. Brain.

[5] Chaudhuri KR (2010). The nondeclaration of nonmotor symptoms of Parkinson’s disease to health care professionals: An international study using the nonmotor symptoms questionnaire. Mov. Disord..

[6] Ponsi G, Scattolin M, Villa R, Aglioti SM (2021). Human moral decision-making through the lens of Parkinson’s disease. npj Parkinson’s Dis..

[7] Seer C, Lange F, Georgiev D, Jahanshahi M, Kopp B (2016). Event-related potentials and cognition in Parkinson’s disease: an integrative review. Neurosci. Biobehav. Rev..

[8] Lenzoni, S., Baker, J., Sumich, A. L. & Mograbi, D. C. New insights into neural networks of error monitoring and clinical implications: a systematic review of ERP studies in neurological diseases. *Rev. Neurosci.* **33**, 161–179 (2021). 10.1515/revneuro-2021-0054 34214387

[9] Pezzetta, R., Wokke, M. E., Aglioti, S. M., & Ridderinkhof, K. R. Doing it wrong: a systematic review on electrocortical and behavioral correlates of error monitoring in patients with neurological disorders. *Neuroscience*10.1016/j.neuroscience.2021.01.027 (2021). 10.1016/j.neuroscience.2021.01.027 33516775

[10] Colzato LS (2012). Dopaminergic modulation of the updating of stimulus–response episodes in Parkinson’s disease. Behav. Brain Res..

[11] Seer C (2017). Dopaminergic modulation of performance monitoring in Parkinson’s disease: An event-related potential study. Sci. Rep..

[12] Wylie SA (2012). Dopamine agonists and the suppression of impulsive motor actions in Parkinson disease. J. Cogn. Neurosci..

[13] Caprio D (2020). Early‐stage Parkinson’s patients show selective impairment in reactive but not proactive inhibition. Mov. Disord..

[14] Trujillo P (2019). Dopamine effects on frontal cortical blood flow and motor inhibition in Parkinson’s disease. Cortex.

[15] Era, V. et al. The dopaminergic system supports flexible and rewarding dyadic motor interactive behaviour in Parkinson’s disease. *Soc. Cogn. Affect. Neurosci*. 10.1093/scan/nsac040 (2022). 10.1093/scan/nsac040 PMC9949502 35674339

[16] van Wouwe NC (2011). Deep brain stimulation of the subthalamic nucleus improves reward-based decision-learning in Parkinson’s disease. Front. Hum. Neurosci..

[17] Clark A (2013). Whatever next? Predictive brains, situated agents, and the future of cognitive science. Behav. Brain Sci..

[18] Friston K, Kiebel S (2009). Predictive coding under the free-energy principle. Philos. Trans. R. Soc. B Biol. Sci..

[19] Schultz W (1998). Predictive reward signal of dopamine neurons. J. Neurophysiol..

[20] Schultz W (2016). Dopamine reward prediction-error signalling: a two-component response. Physiol. Behav..

[21] Cavanagh JF, Zambrano-Vazquez L, Allen JJB (2012). Theta lingua franca: a common mid-frontal substrate for action monitoring processes. Psychophysiology.

[22] Cavanagh JF, Frank MJ (2014). Frontal theta as a mechanism for cognitive control. Trends Cogn. Sci..

[23] Joch M, Hegele M, Maurer H, Müller H, Maurer LK (2017). Brain negativity as an indicator of predictive error processing: The contribution of visual action effect monitoring. J. Neurophysiol..

[24] Ridderinkhof KR, Ullsperger M, Crone EA, Nieuwenhuis S (2004). The role of the medial frontal cortex in cognitive control.. Science.

[25] Koban L, Pourtois G (2014). Brain systems underlying the affective and social monitoring of actions: an integrative review. Neurosci. Biobehav. Rev..

[26] de Bruijn ERA, Schubotz RI, Ullsperger M (2007). An event-related potential study on the observation of erroneous everyday actions. Cogn. Affect. Behav. Neurosci..

[27] van Schie HT, Mars RB, Coles MGH, Bekkering H (2004). Modulation of activity in medial frontal and motor cortices during error observation. Nat. Neurosci..

[28] Era V, Boukarras S, Candidi M (2019). Neural correlates of action monitoring and mutual adaptation during interpersonal motor coordination: Comment on “The body talks: Sensorimotor communication and its brain and kinematic signatures” by G. Pezzulo et al. Phys. Life Rev..

[29] Moreau Q, Candidi M, Era V, Tieri G, Aglioti SM (2020). Midline frontal and occipito-temporal activity during error monitoring in dyadic motor interactions. Cortex.

[30] Pavone EF (2016). Embodying others in immersive virtual reality: electro-cortical signatures of monitoring the errors in the actions of an avatar seen from a first-person perspective. J. Neurosci..

[31] Spinelli G, Tieri G, Pavone EF, Aglioti SM (2018). Wronger than wrong: graded mapping of the errors of an avatar in the performance monitoring system of the onlooker. NeuroImage.

[32] Fusco G (2018). Midfrontal theta transcranial alternating current stimulation modulates behavioural adjustment after error execution. Eur. J. Neurosci..

[33] Jocham G, Ullsperger M (2009). Neuropharmacology of performance monitoring. Neurosci. Biobehav. Rev..

[34] Parker KL, Chen KH, Kingyon JR, Cavanagh JF, Narayanan NS (2015). Medial frontal ~4-Hz activity in humans and rodents is attenuated in PD patients and in rodents with cortical dopamine depletion. J. Neurophysiol..

[35] Ridderinkhof KR, Van Den Wildenberg WP, Segalowitz SJ, Carter CS (2004). Neurocognitive mechanisms of cognitive control: the role of prefrontal cortex in action selection, response inhibition, performance monitoring, and reward-based learning. Brain Cogn..

[36] Holroyd CB, Coles MGH (2002). The neural basis of human error processing: Reinforcement learning, dopamine, and the error-related negativity. Psychol. Rev..

[37] Vocat R, Pourtois G, Vuilleumier P (2008). Unavoidable errors: a spatio-temporal analysis of time-course and neural sources of evoked potentials associated with error processing in a speeded task. Neuropsychologia.

[38] Klein TA, Ullsperger M, Danielmeier C (2013). Error awareness and the insula: links to neurological and psychiatric diseases. Front. Hum. Neurosci..

[39] Orr JM, Carrasco M (2011). The role of the error positivity in the conscious perception of errors. J. Neurosci..

[40] Wessel JR (2018). An adaptive orienting theory of error processing. Psychophysiology.

[41] Overbeek TJM, Nieuwenhuis S, Ridderinkhof KR (2005). Dissociable components of error processing: On the functional significance of the Pe vis-à-vis the ERN/Ne. J. Psychophysiol..

[42] Danielmeier C, Ullsperger M (2011). Post-error adjustments. Front. Psychol..

[43] Di Gregorio F, Maier ME, Steinhauser M (2018). Errors can elicit an error positivity in the absence of an error negativity: Evidence for independent systems of human error monitoring. NeuroImage.

[44] Steinhauser M, Yeung N (2010). Decision processes in human performance monitoring. J. Neurosci..

[45] Falkenstein M, Willemssen R, Hohnsbein J, Hielscher H (2005). Error processing in Parkinson’s disease: The error positivity (Pe). J. Psychophysiol..

[46] De Bruijn ER, Hulstijn W, Verkes RJ, Ruigt GS, Sabbe BG (2004). Drug-induced stimulation and suppression of action monitoring in healthy volunteers. Psychopharmacology.

[47] Ullsperger M, Fischer AG, Nigbur R, Endrass T (2014). Neural mechanisms and temporal dynamics of performance monitoring. Trends Cogn. Sci..

[48] Ridderinkhof KR, Ramautar JR, Wijnen JG (2009). To PE or not to PE: a P3‐like ERP component reflecting the processing of response errors. Psychophysiology.

[49] Ullsperger M, Harsay HA, Wessel JR, Ridderinkhof KR (2010). Conscious perception of errors and its relation to the anterior insula. Brain Struct. Funct..

[50] Wessel JR, Danielmeier C, Ullsperger M (2011). Error awareness revisited: accumulation of multimodal evidence from central and autonomic nervous systems. J. Cogn. Neurosci..

[51] Lerner TN, Holloway AL, Seiler JL (2021). Dopamine, updated: reward prediction error and beyond. Curr. Opin. Neurobiol..

[52] Ehlers CL, Chaplin RI (1992). Long latency event related potentials in rats: the effects of changes in stimulus parameters and neurochemical lesions. J. Neural Transm. Gen. Sect. JNT.

[53] Nieuwenhuis S, Aston-Jones G, Cohen JD (2005). Decision making, the P3, and the locus coeruleus–norepinephrine system. Psychol. Bull..

[54] Beste C (2017). Striosomal dysfunction affects behavioral adaptation but not impulsivity—Evidence from X-linked dystonia-parkinsonism. Mov. Disord..

[55] Singh A, Richardson SP, Narayanan N, Cavanagh JF (2018). Mid-frontal theta activity is diminished during cognitive control in Parkinson’s disease. Neuropsychologia.

[56] Luu P, Tucker DM, Makeig S (2004). Frontal midline theta and the error-related negativity: Neurophysiological mechanisms of action regulation. Clin. Neurophysiol..

[57] Moran, R. J. et al. Alterations in brain connectivity underlying beta oscillations in parkinsonism. *PLoS Comput. Biol*. 10.1371/journal.pcbi.1002124 (2011). 10.1371/journal.pcbi.1002124 PMC3154892 21852943

[58] Pezzetta, R., Nicolardi, V., Tidoni, E. & Aglioti, S. M. Error, rather than its probability, elicits specific electrocortical signatures: a combined EEG-immersive virtual reality study of action observation. *J. Neurophysiol.* **120**, 1107–1118 (2018). 10.1152/jn.00130.2018 29873613

[59] Falkenstein M (2001). Action monitoring, error detection, and the basal ganglia: an ERP study. NeuroReport.

[60] Eckart C, Fuentemilla L, Bauch EM, Bunzeck N (2014). Dopaminergic stimulation facilitates working memory and differentially affects prefrontal low theta oscillations. NeuroImage.

[61] Singh A (2021). Timing variability and midfrontal~4 Hz rhythms correlate with cognition in Parkinson’s disease. npj Parkinson’s Dis..

[62] KIM MS (2006). Neuropsychological correlates of error negativity and positivity in schizophrenia patients. Psychiatry Clin. Neurosci..

[63] Willemssen R, Müller T, Schwarz M, Hohnsbein J, Falkenstein M (2008). Error processing in patients with Parkinson’s disease: the influence of medication state. J. Neural Transm..

[64] Singh A (2018). Oscillatory activity in the cortico‐basal ganglia‐thalamic neural circuits in Parkinson’s disease. Eur. J. Neurosci..

[65] Kühn AA (2004). Patterns of abnormal motor cortex excitability in atypical parkinsonian syndromes. Clin. Neurophysiol..

[66] Silberstein P (2005). Cortico-cortical coupling in Parkinson’s disease and its modulation by therapy. Brain.

[67] Alberico SL, Cassell MD, Narayanan NS (2015). The vulnerable ventral tegmental area in Parkinson’s disease. Basal Ganglia.

[68] Moreau, Q., Tieri, G., Era, V., Aglioti, S. M. & Candidi, M. The performance monitoring system is attuned to others’ actions during dyadic motor interactions. *Cereb. Cortex* **33**, 222–234 (2023). 10.1093/cercor/bhac063 35203090

[69] Solié C, Girard B, Righetti B, Tapparel M, Bellone C (2022). VTA dopamine neuron activity encodes social interaction and promotes reinforcement learning through social prediction error. Nat. Neurosci..

[70] Boukarras S (2022). Midfrontal theta transcranial alternating current stimulation facilitates motor coordination in Dyadic Human–Avatar interactions. J. Cogn. Neurosci..

[71] Sauseng P, Klimesch W, Schabus M, Doppelmayr M (2005). Fronto-parietal EEG coherence in theta and upper alpha reflect central executive functions of working memory. Int. J. Psychophysiol..

[72] Chen KH (2016). Startle habituation and midfrontal theta activity in Parkinson disease. J. Cogn. Neurosci..

[73] Bosboom JLW, Stoffers D, Wolters EC (2004). Cognitive dysfunction and dementia in Parkinson’s disease. J. Neural Transm..

[74] Iijima M, Osawa M, Iwata M, Miyazaki A, Tei H (2000). Topographic mapping of P300 and frontal cognitive function in Parkinson’s disease. Behav. Neurol..

[75] Maier ME, Di Gregorio F, Muricchio T, Di Pellegrino G (2015). Impaired rapid error monitoring but intact error signaling following rostral anterior cingulate cortex lesions in humans. Front. Hum. Neurosci..

[76] Ullsperger M, Von Cramon DY, Müller NG (2002). Interactions of focal cortical lesions with error processing: evidence from event-related brain potentials. Neuropsychology.

[77] Spinelli, G., Pezzetta, R., Canzano, L., Tidoni, E., & Aglioti, S. M. Brain Dynamics of action monitoring in higher-order motor control disorders: the case of apraxia. *Eneuro* **9**, (2022). 10.1523/ENEURO.0334-20.2021 PMC8896553 35105660

[78] Mathewson KJ, Dywan J, Segalowitz SJ (2005). Brain bases of error-related ERPs as influenced by age and task. Biol. Psychol..

[79] Nieuwenhuis S, Richard Ridderinkhof K, Blom J, Band GPH, Kok A (2001). Error-related brain potentials are differentially related to awareness of response errors: evidence from an antisaccade task. Psychophysiology.

[80] Thurm, F., Li, S. C., & Hämmerer, D. Maturation- and aging-related differences in electrophysiological correlates of error detection and error awareness. *Neuropsychologia*10.1016/j.neuropsychologia.2020.107476 (2020). 10.1016/j.neuropsychologia.2020.107476 PMC7322543 32360297

[81] Wang L, Gu Y, Zhao G, Chen A (2020). Error-related negativity and error awareness in a Go/No-go task. Sci. Rep..

[82] Weller L, Schwarz KA, Kunde W, Pfister R (2018). My mistake? Enhanced error processing for commanded compared to passively observed actions. Psychophysiology.

[83] Trujillo LT, Allen JJB (2007). Theta EEG dynamics of the error-related negativity. Clin. Neurophysiol..

[84] van Driel J, Ridderinkhof KR, Cohen MX (2012). Not all errors are alike: theta and alpha EEG dynamics relate to differences in error-processing dynamics. J. Neurosci..

[85] Koelewijn T, van Schie HT, Bekkering H, Oostenveld R, Jensen O (2008). Motor-cortical beta oscillations are modulated by correctness of observed action. NeuroImage.

[86] Jurkiewicz MT, Gaetz WC, Bostan AC, Cheyne D (2006). Post-movement beta rebound is generated in motor cortex: evidence from neuromagnetic recordings. NeuroImage.

[87] Pfurtscheller G, Lopes da Silva FH. Event-related EEG/MEG synchronization and desynchronization: basic principles. *Clin Neurophysiol* **110**, 1842–1857 (1999). 10.1016/s1388-2457(99)00141-8 10576479

[88] Torrecillos F, Alayrangues J, Kilavik BE, Malfait N (2015). Distinct modulations in sensorimotor postmovement and foreperiod -band activities related to error salience processing and sensorimotor adaptation. J. Neurosci..

[89] Viñales, L., Procyk, E., & Quilodran, R. Feedback-related potentials and oscillations during trial and error learning in Parkinson’s disease. *BioRxiv* 1–25 10.1101/2021.04.05.438433 (2021).

[90] Engel AK, Fries P (2010). Beta-band oscillations-signalling the status quo?. Curr. Opin. Neurobiol..

[91] Babiloni C (2016). Alpha, beta and gamma electrocorticographic rhythms in somatosensory, motor, premotor and prefrontal cortical areas differ in movement execution and observation in humans. Clin. Neurophysiol..

[92] Babiloni C (2017). Frontal functional connectivity of electrocorticographic delta and theta rhythms during action execution versus action observation in humans. Front. Behav. Neurosci..

[93] Oswal A, Brown P, Litvak V (2013). Synchronized neural oscillations and the pathophysiology of Parkinson’s disease. Curr. Opin. Neurol..

[94] Doyle LMF (2005). Levodopa-induced modulation of subthalamic beta oscillations during self-paced movements in patients with Parkinson’s disease. Eur. J. Neurosci..

[95] Pollok B (2013). Increased SMA–M1 coherence in Parkinson’s disease—pathophysiology or compensation?. Exp. Neurol..

[96] Villa R, Tidoni E, Porciello G, Aglioti SM (2018). Violation of expectations about movement and goal achievement leads to Sense of Agency reduction. Exp. Brain Res..

[97] Villa R, Tidoni E, Porciello G, Aglioti SM (2021). Freedom to act enhances the sense of agency, while movement and goal-related prediction errors reduce it. Psychol. Res..

[98] Villa, R., Ponsi, G., Scattolin, M., Panasiti, M. S., & Aglioti, S. M. Social, affective, and non-motoric bodily cues to the sense of agency: a systematic review of the experience of control. *Neurosci. Biobehav. Rev*. 104900 10.1016/j.neubiorev.2022.104900 (2022). 10.1016/j.neubiorev.2022.104900 36202254

[99] Porras Garcia B (2019). Is this my own body? Changing the perceptual and affective body image experience among college students using a new virtual reality embodiment-based technique. J. Clin. Med..

[100] Tieri G, Morone G, Paolucci S, Iosa M (2018). Virtual reality in cognitive and motor rehabilitation: facts, fiction and fallacies. Expert Rev. Med. devices.

[101] Ozkan, D. G., & Pezzetta, R. Predictive monitoring of actions, EEG recordings in virtual reality. *J. Neurophysiol*. 10.1152/jn.00825.2017 (2018). 10.1152/jn.00825.2017 29357471

[102] Mattia M (2012). Stop-event-related potentials from intracranial electrodes reveal a key role of premotor and motor cortices in stopping ongoing movements. Front. Neuroeng..

[103] Calabresi P, Picconi B, Parnetti L, Di Filippo M (2006). A convergent model for cognitive dysfunctions in Parkinson’s disease: the critical dopamine-acetylcholine synaptic balance. Lancet Neurol..

[104] Van Nuland AJ (2021). Effects of dopamine on reinforcement learning in Parkinson’s disease depend on motor phenotype. Brain.

[105] Krigolson OE, Holroyd CB (2007). Hierarchical error processing: different errors, different systems. Brain Res..

[106] Friston, K. J. et al. Dopamine, affordance and active inference. *PLoS Comput. Biol*. 10.1371/journal.pcbi.1002327 (2012). 10.1371/journal.pcbi.1002327 PMC3252266 22241972

[107] Masina, F. et al. Disconnection from prediction: a systematic review on the role of right temporoparietal junction in aberrant predictive processing. *Neurosci. Biobehav. Rev.* 104713 10.1016/j.neubiorev.2022.104713 (2022). 10.1016/j.neubiorev.2022.104713 35636560

[108] Campbell JID, Thompson VA (2012). MorePower 6.0 for ANOVA with relational confidence intervals and Bayesian analysis. Behav. Res. Methods.

[109] Fahn, S., Elton, R & Members of the UPDRS Development Committee. in *Recent Developments in Parkinson’s Disease* (eds. Fahn S, Marsden CD, Calne DB, Lieberman A) 153–163 (Macmillan Health Care Information, 1987).

[110] Dubois B (2007). Diagnostic procedures for Parkinson’s disease dementia: recommendations from the movement disorder society task force. Mov. Disord..

[111] Cruz-Neira, C., Sandin, D. J., & DeFanti, T. A. Surround-screen projection-based virtual reality: the design and implementation of the CAVE. In Proceedings of the 20th annual conference on Computer graphics and interactive techniques pp. 135–142 (1993).

[112] Tecchia, F. et al. I’m in VR!: using your own hands in a fully immersive MR system. In Proceedings of the 20th ACM Symposium on Virtual Reality Software and Technology pp. 73–76 (2014).

[113] Langston JW (1992). Core assessment program for intracerebral transplantations (CAPIT). Mov. Disord..

[114] Casula, E. P. et al. Feeling of ownership over an embodied avatar’s hand brings about fast changes of fronto-parietal cortical dynamics. *J. Neurosci*. 10.1523/jneurosci.0636-21.2021 (2021). 10.1523/JNEUROSCI.0636-21.2021 PMC8805621 34862188

[115] Fusaro M, Tieri G, Aglioti SM (2019). Influence of cognitive stance and physical perspective on subjective and autonomic reactivity to observed pain and pleasure: an immersive virtual reality study. Conscious. Cogn..

[116] Fusco G, Tieri G, Aglioti SM (2020). Visual feedback from a virtual body modulates motor illusion induced by tendon vibration. Psychol. Res..

[117] Tieri G, Tidoni E, Pavone EF, Aglioti SM (2015). Body visual discontinuity affects feeling of ownership and skin conductance responses. Sci. Rep..

[118] Tieri G, Tidoni E, Pavone EF, Aglioti SM (2015). Mere observation of body discontinuity affects perceived ownership and vicarious agency over a virtual hand. Exp. Brain Res..

[119] Hoehn, M. M., & Yahr, M. D. (1969). in *Third Symposium on Parkinson’s Disease*, 274–280. (Livingstone, 1969).

[120] Eggermont LH (2010). Lower-extremity function in cognitively healthy aging, mild cognitive impairment, and Alzheimer’s disease. Arch. Phys. Med. Rehabil..

[121] Jung TP (2000). Removal of eye activity artifacts from visual event-related potentials in normal and clinical subjects. Clin. Neurophysiol..

[122] Drisdelle BL, Aubin S, Jolicoeur P (2017). Dealing with ocular artifacts on lateralized ERPs in studies of visual‐spatial attention and memory: ICA correction versus epoch rejection. Psychophysiology.

[123] Pontifex MB (2010). On the number of trials necessary for stabilization of error-related brain activity across the life span. Psychophysiology.

[124] Tadel, F., Baillet, S., Mosher, J. C., Pantazis, D., & Leahy, R. M. Brainstorm: a user-friendly application for MEG/EEG analysis. *Comput. Intell. Neurosci.*10.1155/2011/879716 (2011). 10.1155/2011/879716 PMC3090754 21584256

[125] R Core Team. *R: A Language and Environment for Statistical Computing*. (R Foundation for Statistical Computing, 2013).

[126] Arcara, G. & Petrova, A. *erpR: event-related potentials (ERP) analysis, graphics and utility functions (version 0.2.0)*. https://rdrr.io/cran/erpR/. (2014).

[127] Luck, S. J. Event-related potentials. In Cooper, H., Camic, P. M., Long, D. L., Panter, A. T., Rindskopf, D., & Sher K. J. (Eds.), APA handbook of research methods in psychology, Foundations, planning, measures, and psychometrics. American Psychological Association. Vol. 1. (pp. 523–546). 10.1037/13619-028 (2012).

[128] Formica, S., González-García, C., Senoussi, M., Marinazzo, D., & Brass, M. Theta-phase connectivity between medial prefrontal and posterior areas underlies novel instructions implementation. *Eneuro* 9 10.1523/ENEURO.0225-22.2022 (2022). 10.1523/ENEURO.0225-22.2022 PMC9374157 35868857

[129] Maris E, Oostenveld R (2007). Nonparametric statistical testing of EEG-and MEG-data. J. Neurosci. Methods.

[130] Van Dinteren R, Huster RJ, Jongsma MLA, Kessels RPC, Arns M (2018). Differences in cortical sources of the event-related P3 potential between young and old participants indicate frontal compensation. Brain Topogr..

[131] Kappenman ES, Luck SJ (2016). Best practices for event-related potential research in clinical populations. Biol. Psychiatry. Cogn. Neurosci. Neuroimaging.

[132] Dienes Z (2014). Using Bayes to get the most out of non-significant results. Front. Psychol..

[133] Love J (2019). JASP: Graphical statistical software for common statistical designs. J. Stat. Softw..

[134] Cohen, Mike X. Analyzing neural time series data: theory and practice. MIT press, 2014.

[135] Malfait N (2010). fMRI activation during observation of others’ reach errors. J. Cogn. Neurosci..

[136] Fusco G, Fusaro M, Aglioti SM (2022). Midfrontal-occipital θ-tACS modulates cognitive conflicts related to bodily stimuli. Soc. Cogn. Affect. Neurosci..

[137] Cohen, M. X. Comparison of different spatial transformations applied to EEG data: A case study of error processing. *Int J Psychophysiol* **97**, 245–257 (2015). 10.1016/j.ijpsycho.2014.09.013 25455427

[138] Ullsperger M, Von Cramon DY (2004). Neuroimaging of performance monitoring: error detection and beyond. Cortex.

[139] Giovagnoli AR (1996). Trail making test: normative values from 287 normal adult controls. Ital. J. Neurol. Sci..

[140] Measso G (1993). The mini‐mental state examination: normative study of an Italian random sample. Dev. Neuropsychol..

[141] Costa A (2013). Mini mental Parkinson test: standardization and normative data on an Italian sample. Neurol. Sci..

